# Genome-Wide Identification, Phylogeny and Expressional Profiles of Mitogen Activated Protein Kinase Gene Family in *Blakeslea trispora*

**DOI:** 10.3390/ijms26104789

**Published:** 2025-05-16

**Authors:** Xin Ge, Yue Cui, Yanan Zhang, Jianlin Li, Ping Wang, Yan Zheng, Qi Xin

**Affiliations:** 1 School of Life Science, Hebei University, Baoding 071002, China; gexin@hbu.edu.cn (X.G.);; 2 Key Laboratory of Microbial Diversity Research and Application of Hebei Province, Baoding 071002, China; 3 Engineering Research Center of Microbial Breeding and Conservation of Hebei Province, Baoding 071002, China

**Keywords:** MAPK gene family, mating and blue light responses, protein-protein interaction, expression patterns

## Abstract

In eukaryotes, the mitogen-activated protein kinase (MAPK) cascade pathway is a highly conserved cell signaling mechanism that is essential for stress response, growth, and development. MAPK cascade genes have currently been identified and characterized in a wide range of fungi, although they have not been fully understood in early divergent fungal lineages like the Mucoromycota, which contains Mucoromycotina, Glomeromycotina, and Mortierellomycotina. In this study, a genome-wide investigation of *Blakeslea trispora* (Mucorales, Choanephoraceae) revealed a total of 19 MAPK cascade genes, including 9 BtMAPKKKs, 4 BtMAPKKs, and 6 BtMAPKs genes. Using phylogenetic analysis, it was found that the kinase domain sequences and motif composition of the three MAPK, MAPKK, and MAPKKK lineages are substantially conserved in fungi. Whole genome duplication analysis indicated that *B. trispora* has four and nine duplication pairs in the *MAPK* and *MAPKKK* genes, respectively, which are expanded by segmental replication events. BtHog2, the orthologous protein of Hog1, exhibits a substantial rise in transcription levels under blue light irradiation, indicating its function in light signal response and transduction. Several sets of interacting protein pairs were found using molecular docking analysis and yeast two-hybrid assay, providing a comprehensive MAPK cascade signaling network in *B. trispore*. Furthermore, MAPK cascade proteins show varying transcription levels in response to blue light and sex hormone stimulation, as well as variable treatment duration. *BtMAPKKK*9 and *BtBck*1 are strongly induced during sexual interaction, indicating their involvement in the response to trisporic acid and the subsequent alterations in hyphal cell wall structure. These findings shed light on the evolution of MAPK cascade genes and the functional mechanisms underlying MAPK cascade genes in response to light and sex hormone signaling pathways in *B. trispore*.

## 1. Introduction

*Blakeslea trispora*, an ideal natural carotenoid producer, has gained much attention due to the biosynthesis of high value-added products such as β-carotene and lycopene. Previous studies have primarily focused on the fermentation production of carotenoids and the metabolic regulation of biosynthetic pathways [1,2,3]. Currently, it is believed that sex interaction of plus/minus strains (sexual reproduction), light stimulation, and oxidative stress generated during the fermentation process can enhance carotenoid production [4,5,6,7]. However, these studies are insufficient, and further research is needed to explore the molecular mechanisms influencing carotenoid synthesis.

The mitogen-activated protein kinase (MAPK) signaling pathway is widely present in eukaryotes and exhibits high conservation throughout evolution. Generally, this cascade consists of three core kinases: MAPK, MAPK kinase (MAPKK), and MAPKK kinases (MAPKKK) [8]. Upon perception of extracellular stimuli by a membrane receptor, the MAPK pathway initiates signal translation into cellular responses through phosphorylation events involving MAPKKKs and subsequent activation of MAPKKs. Ultimately, activated MAPKs trigger downstream transcription factors that regulate diverse cellular functions [9].

The presence of MAPK pathways has been identified in various fungi, where they play roles in regulating cell growth, development, oxidative pressure response, osmotic pressure response, and pathogenicity [10,11,12]. The most information available about the fungal MAPK pathways comes from studies on the baker’s yeast *Saccharomyces cerevisiae*. Cell wall integrity (CWI) pathway, the high osmolarity glycerol (HOG) pathway, Kss/Fus3 cascade, and Mpk1 and Smk1 pathways are the major MAPK pathways described in *S. cerevisiae* and many other fungi belonging to different divisions, such as *Cryptococcus neoformans*, *Fusarium oxysporum*, and *Aspergillus nidulans* [9,13,14,15]. Although the MAPK signaling pathways are highly conserved in eukaryotic cells, as the best-understood pathways, orthologous signaling modules in various fungi have their distinct characteristics. For instance, the cell wall integrity (CWI) pathway plays a fundamental role in maintaining the stability of the cell wall under adverse environmental conditions, and it is essential for virulence in many phytopathogenic fungi [16]. The HOG pathway responds to changes in external osmotic stress by regulating intracellular glycerol synthesis *in S. cerevisiae.* In *Rhizophagus irregularis*, the HOG1-MAPK pathway has been demonstrated to be essential for arbuscule development, plant drought tolerance, and conserved osmoregulatory mechanisms underpinning mutualistic symbiosis [17,18,19]. It has also been proved to sense and respond to nutrients in *N. crassa* and be involved in responding to oxidative, heat, and other environmental stresses [11]. The Fus3 pathway is known as the pheromone response module, which was initially characterized in *S. cerevisiae* and was shown to be involved in mating and cell fusion. Kss1 shares a partially overlapping function in mating with Fus3. It can also be activated to respond to nutrient starvation, whereby filamentation or invasive growth is regulated [20,21].

Currently, MAPK, MAPKK, and MAPKKK proteins and their cascade pathways in numerous fungi have been characterized by molecular or biochemical approaches, and there are still more that have been predicted using bioinformatics [22,23]. However, the identity and functions of the MAPK cascade in *B. trispora* remain largely unknown until now. Previous research has shown that reactive oxygen species (ROS) are generated during the fermentation process, resulting in an oxidative stress response that significantly increases carotene yield [6,24,25]. It has been determined that three photoreceptors, BtWC-1A, BtWC-1B, and BtWC-1C, are involved in phototropic and photo-induced carotenoid biosynthesis [26]. It is yet unknown, though, if the light sensor can trigger the MAPK cascade pathway and initiate the transcriptional response of genes downstream. Likewise, it is worthwhile to conduct extensive research into whether the sex hormones generated by plus/minus strains mating correlate with the MAPK cascade pathway.

Therefore, to advance the understanding of carotenoid biosynthesis regulation in *B. trispora*, this study aimed to systematically investigate the MAPK cascade signaling pathways in response to light stimuli and sex pheromone signals. Leveraging the genomic resources of *B. trispora*, we conducted genome-wide identification and annotation of MAPK cascade core components (MAPK, MAPKK, and MAPKKK), followed by phylogenetic reconstruction, conserved motif analysis, and adaptive evolution profiling to decipher their evolutionary divergence and functional specialization. Concurrently, transcriptional profiling and yeast two-hybrid assays were employed to unravel the roles of the MAPK cascade in responding to light and sex hormone signals, as well as to investigate potential cross-talk between these signaling pathways under the two stress conditions. By integrating bioinformatic predictions with experimental validation, this work establishes a framework for understanding how MAPK signaling networks modulate carotenoid production in *B. trispora*, offering novel insights for targeted metabolic engineering strategies in industrial fungal biotechnology.

## 2. Results

### 2.1. Identification of MAPK, MAPKK, and MAPKKK Family Genes in B. trispora

To fully clarify the evolutionary relationship of putative MAPK, MAPKK, and MAPKKK family genes in *B. trispora* as well as their functional significance in signal transduction, a blast search was performed in the *B. trispora* protein database using *S. cerevisiae* MAPK cascade protein sequences as queries. Consequently, we identified a total of 19 MAPK cascade proteins comprising 6 MAPKs, 4 MAPKKs, and 9 MAPKKKs. Based on the corresponding homologues in other species, the predicted proteins were given the names BtMAPK1-6, BtMAPKK1-4, and BtMAPKKK1-9. The Ser/Thr kinase domain is present in all predicted proteins, yet the conserved phosphorylation site varies slightly. In detail, BtMAPKs harbor the specific conserved signature motif TxY, BtMAPKKs possess the conserved [S/T]xxx[S/T] sites, and BtMAPKKKs share the G[S/T][V/P][F/M][W/Y]M[A/S]PEV signature.

**Figure 1:**
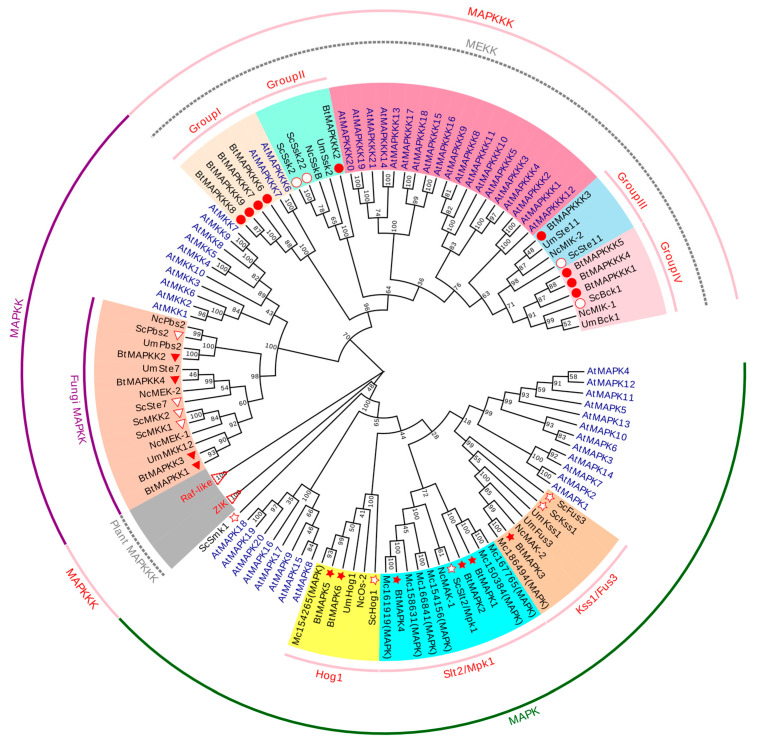
Phylogenetic analysis of MAPK cascade proteins from *B. trispora*, *A*. *thaliana,* and several representative fungi. The neighbor-joining tree was constructed using the MEGAX program based on full-length protein sequences of a total of 166 MAPK cascade proteins. The bootstrap value was set to 1000 replicates. To identify the species of origin for each protein, a species acronym is included before the protein name: Bt, *B. trispora*; At, *A. thaliana*; Sc, *S. cerevisiae*; Nc, *N. crassa*; Mc, *M. circinelloides*; Um, *U. maydis*. Raf-like and ZIK clades of the MEKK were marked with triangles since no fungal proteins clustered into them. The identified MAPKs, MAPKKs, and MAPKKKs in *B. trispora* were marked by solid stars, triangles, and circles, respectively. Meanwhile, the known MAPKs, MAPKKs, and MAPKKKs in *S. cerevisiae* were marked by blank stars, triangles, and circles, respectively. Different groups were represented by different colors.

Furthermore, the physicochemical properties of the identified MAPK cascade proteins were systematically characterized in Table 1. Following phylogenetic analysis (Figure 1), the 19 proteins were renamed along with their corresponding homologous gene symbols in *S. cerevisiae*. The amino acid lengths of MAPK cascade proteins exhibited substantial variation, ranging from 259 (MAPKKK6) to 1327 amino acids (BtSsk2). Their molecular weight varied from 28.5 kDa (BtMAPKKK6) to 151.5 kDa (BtSsk2), while the isoelectric point (pI) varied between 5.16 (BtMpk1) and 9.03 (BtMAPKKK9). Hydrophobicity analysis revealed that all analyzed MAPK cascade proteins exhibited hydrophilic characteristics and lacked transmembrane domains.

### 2.2. Phylogenetic Analysis of MAPK, MAPKK, and MAPKKK Family Genes in B. trispora

To further evaluate the evolutionary relationship of *Bt*MAPK cascade proteins, it is necessary to construct a phylogenetic tree based on full-length amino acid sequences of the 19 identified BtMAPK cascade genes and the corresponding genes in *S. cerevisiae*, *Arabidopsis thaliana*, *Neurospora crassa*, *Ustilago maydis(DC)Corola*, and *M. circinelloides*.

Phylogenetic analysis of the MAPK cascade proteins, using full-length sequences from 166 proteins across six species, revealed that *B. trispora* MAPK cascades exhibit closer homology to fungal orthologs (e.g., *S. cerevisiae*, *M. circinelloides*) than to plant counterparts (*A. thaliana*). The six BtMAPKs clustered into three evolutionarily conserved subfamilies: BtMpk1, BtMpk2, and BtMpk3 are members of the Slt2/Mpk1 group; BtFus3 is grouped within the Kss1/Fus3 group; and BtHog1 and BtHog2 are clustered within the Hog1 subfamily. Notably, all BtMAPKs showed high sequence similarity to the McMAPKs derived from *M. circinelloides*, underscoring conserved fungal signaling modules. Similarly, BtMAPKKs clustered exclusively with fungal lineages, diverging sharply from *A. thaliana* MAPKKs. The nine BtMAPKKKs segregated into four major groups (I, II, III, and IV), mirroring patterns observed in other species. Group I (BtMAPKKK6–9) is closely related to *A. thaliana*’s MAPKKK5 and MAPKKK6, suggesting ancestral functional overlap. Group II, which comprises the extensively studied proteins ScSsk22 and ScSsk2, includes BtSsk2. BtSte11 is grouped with ScSte11 protein in Group III. BtBck1, BtBck2, and BtBck3, which are found in Group VI, are quite similar to *S. cerevisiae*’s ScBck1. Strikingly, fungal MAPK cascades formed phylogenetically distinct clades despite functional parallels with plant kinases, highlighting lineage-specific evolutionary trajectories.

### 2.3. Gene and Protein Structures Analysis of MAPK, MAPKK, and MAPKKK Family Genes in B. trispora

**Figure 2:**
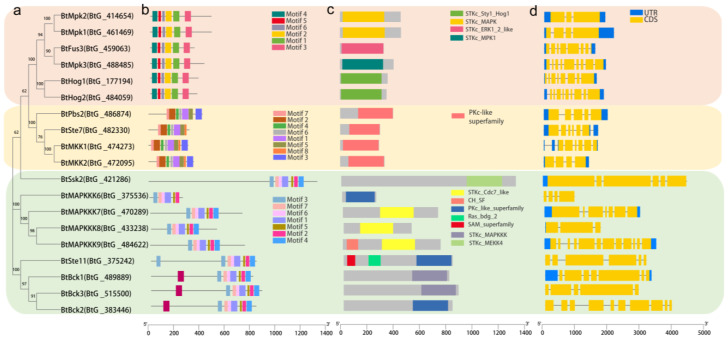
Phylogenetic relationships (**a**), conserved motif location (**b**), domain analysis (**c**), and intron-exon structures (**d**) of MAPK cascade family genes in *B. trispora*. The phylogenetic tree was constructed based on the full-length sequences of BtMAPK cascade family genes. Scanning of the protein sequences for the conserved motifs and domains was performed. The yellow and blue boxes in the exon/intron organization indicated coding sequence and untranslated region, respectively, and thin black lines indicated introns.

Understanding the evolution of gene families is frequently aided by gene structure research, particularly with regard to exon/intron composition, which also offers evidence for their significance in genome functional diversity. As a result, we further performed the exon/intron organization of the BtMAPK cascade genes. The findings show that while introns appear in all of these genes, there exists a considerable diversity in the quantity of these introns (Figure 2d). For instance, the BtMAPK and BtMAPKK gene families have intron counts ranging from 2 to 8 and 1 to 5, respectively, whereas the BtMAPKKK gene family has a larger degree of variability (2–12) in its intron count, clearly indicating a divergence in gene structure. Additionally, the conserved domains in the BtMAPK cascade genes were identified and analyzed by searching against the InterProScan database (Figure 2c). It has been demonstrated that protein kinase domains (Pkinase) are essential for mediating phosphorylation, which is a common occurrence in the majority of biological processes. The BtMAPKs cascade family members all exhibited a high degree of conservation of the domain (Figure 2b). It is important to note that the Ras_bdg_2 domains found in BtSte11, BtBck2, and BtBck3 interact with RasG protein to enable transmembrane receptors to receive and transmit environmental signals. Furthermore, SAM_2 (sterility α motif) domains, which are involved in a number of biological processes such as cell signaling, transcriptional translation regulation, and protein-protein interaction-based developmental process regulation, are present in both BtSte11 and BtBck3. In particular, a CH domain, the function of which has not been reported so far, was first identified in BtMAPKKK9. In general, the identification as well as comprehensive analysis of the conserved domains can facilitate to reveal the specific biological functions for these kinases.

### 2.4. Multiple Sequence Alignment and Conserved Motif Analysis of MAPK, MAPKK, and MAPKKK Family Genes in B. trispora

Conserved motif analysis was performed for each of the MAPK cascade gene families, and the results revealed that they are highly conserved. There are six conserved motifs found in all BtMAPKs and eight motifs in all BtMAPKKs. In nine BtMAPKKKs, eight conserved motifs were identified. Seven of the eight conserved motifs were present across all BtMAPKKKs, whereas motif 8 was specific to BtBck1, BtBck2, and BtBck3. These motifs are likewise quite conserved, as seen by the motif distribution, which is ordered from N-terminal to C-terminal. Table S1 provides extensive information about the eight discovered motifs that were subjected to conserved sequence analysis in this study.

**Figure 3:**
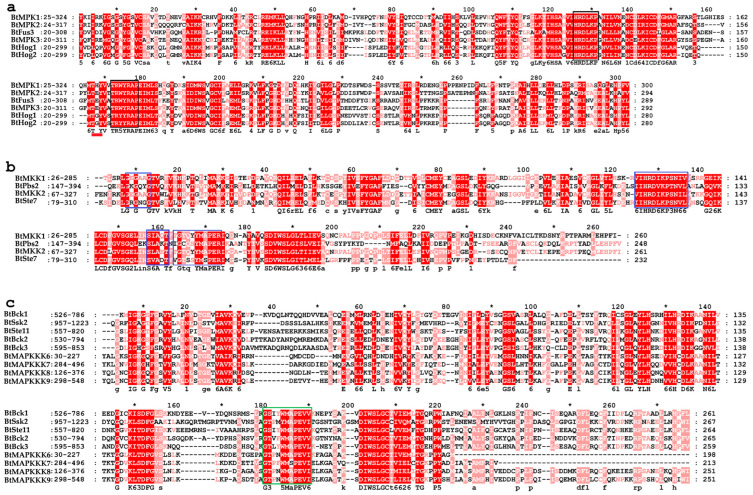
The sequence alignment of serine/threonine kinase domains of MAPK cascade proteins in *B. trispora*. (**a**) BtMAPKs, (**b**) BtMAPKKs, (**c**) BtMAPKKKs. Alignment was performed using ClustalW. The highlighted part showed the conserved signature motif. A red bold line marked the activation loop motifs T-x-Y. The black boxes showed the conserved domain HRDLKPN in the N-terminal and TRWYRAP in the C-terminal. The blue boxes are the conserved motifs in BtMAPKKs, and the third one is the (S/T)-X3-5-(S/T) motif of the activation loop region. The green box indicated a conserved kinase domain G(T/S)Px(W/Y/F)MAPEV in the BtMAPKKK family. * marks midpoint positions between every 20 amino acid residues (i.e., 10, 30, 50...).

To elucidate the conserved characteristics of MAPKKK, MAPKK, and MAPK protein family members of *B. trispora*, phylogenetic analysis and multiple sequence alignment were performed on their kinase domains, respectively (Figure 3). The findings showed that members of the BtMAPKs protein family exhibited great conservation, as did the active sites previously described.

In particular, the TGY/THY/TEY sites, which are the conserved activation loop region of BtMAPKs and phosphorylated by MAPKKs, were found between two conserved domains. The motif 2 included the N-terminal conserved domain of TxY termed HRDLKPS, whereas motif 1 had a C-terminal significant highly conserved domain featuring the sequence “TRWYRAP”. Moreover, BtMAPKs were found to possess a CD domain in their C-terminal region (motif 3), which is defined as YHDPTDEP and functions as a docking site for MAPKKs. Each of the BtMAPKKs was shown to contain the conserved lysine (K) and aspartate (D) residues within the active site motif D (I/V/L) K and a highly conserved phosphorylation target site S-xxx-T within the activation loop. The motifs annotation showed that motif 1 had the phosphorylation target site S-xxx-T and signature TGTQYYMAPER, which were conserved in the catalytic domain, in addition to the active-site signature IIHRDIKPSNILV of serine/threonine protein kinases. The protein kinase ATP-binding signature, which required a glycine-rich loop (GxGxYG) for ATP binding, was also present in motif 7. Each BtMAPKKK, according to multiple alignment, shares the highly conserved signature “-G(T/S)x(W/Y)MAPE(L/V)-” (motif 5) among MAPKKK family kinases. Motif annotation showed that motif 1 contained not only a protein kinase ATP-binding site “BIVHRDJKAANILTT” but also a tyrosine kinase phosphorylation site. In addition, motif 3 contained the serine/threonine protein kinase active site.

### 2.5. Tertiary Structure and Protein-Protein Interaction Prediction of MAPK, MAPKK, and MAPKKK Proteins in B. trispora

**Figure 4:**
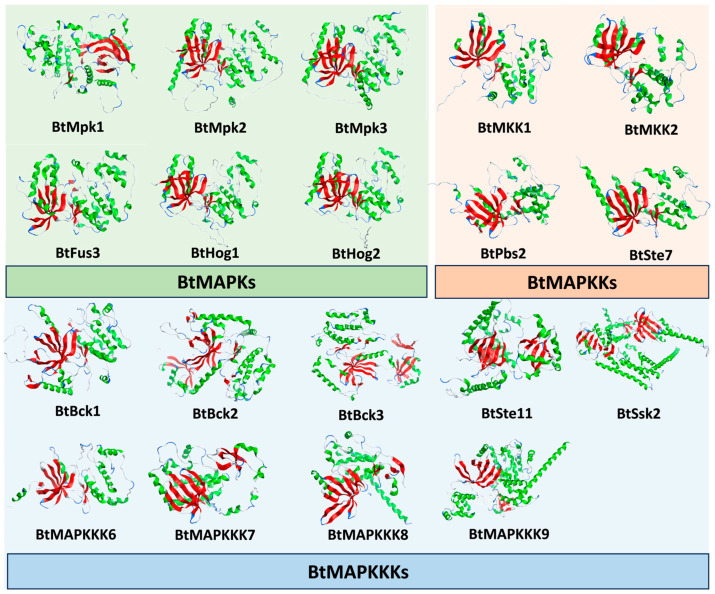
Three-dimensional structures of BtMAPKs, BtMAPKKs, and BtMAPKKKs predicted by AlphaFold. The green region and the red region indicate the alpha helices and beta sheets individually; light blue colored regions depict the turns, and the grey color are the loops.

To date, the copies of MAPK cascade protein genes have been found to be more abundant in the genome of Mucoromycetes than other fungi, such as *S. cerevisiae* and *N. crassa*. Even as a model species for signaling in Mucoromycetes, *P. blakesleeanus* has not been extensively studied regarding its signaling. In order to gain a comprehensive insight into the MAPK signaling network in *B. trispora*, bioinformatics can provide some theoretical evidence for our understanding of the interactions among the MAPK proteins. However, due to the unavailability of crystallographic structures for *B. trispora* MAPKs, MAPKKs, and MAPKKs, the alpha Fold Protein Structure Prediction Database was employed to determine the 3D structure of the MAPKs. During the prediction process based on the full-length sequence of the proteins, some of the protein structures exhibited long irregular regions. Consequently, we truncated the protein sequences based on the initial structure prediction results and performed the 3D structure modeling once more, yielding a total of 19 structures of the selected proteins (Figure 4), which were subsequently used for the in silico prediction of protein–protein interactions.

For understanding the molecular interaction features of a protein, it is necessary to have the information about the protein’s three-dimensional (3D) structure. The docking of BtMAPKKKs and BtMAPKs with BtMAPKKs was performed following ZDOCK and RDOCK programs. The potential protein-protein interaction was predicted using the E_RDOCK scores found in the RDOCK output results. Setting-15 as the E_RDOCK score threshold value will result in better docking poses since a lower E_RDOCK score indicates a stronger interaction. The output values of docking studies, including BtMAPKs and BtMAPKKs modules and BtMAPKKs and BtMAPKKKs modules, were shown in Table S2. A total of 28 pairs of interacting proteins were obtained among all the prediction results. BtMpk1, BtBck1, BtSte11, BtMAPKKK9, BtMAPKKK7, BtHog2, and BtBck2 were the seven potential interaction partners for BtMKK1 (Table 2). The lowest E_RDOCK score was recorded for BtMKK1-BtMpk1, followed by BtMKK1- BtBck1. Among the docking positions of BtPbs1-BtMAPKs and BtPbs1-BtMAPKKKs, three interacting BtMAPKs were BtFus3, BtHog1, and BtHog2, while four interacting BtMAPKKKs were BtSsk2, BtSte11, BtMAPKKK7, and BtMAPKKK9. The best docking position was BtPbs1-BtFus3. BtMKK2 is expected to interact with six proteins, including BtMpk1, BtFus3, BtHog1, BtSsk2, BtMAPKKK6, and BtMAPKKK7. In addition, eight predictions of BtSte7 interacting with BtMAPKs and BtMAPKKKs were BtSsk2, BtHog1, BtMAPKKK9, BtMAPKKK7, BtSte11, BtMpk1, BtMpk3, and BtHog2. Among the eight interacting pairs of proteins, BtSte7-BtSsk2 is the top putative interacting module.

### 2.6. Study of Interactions Involving B. trispora MAPK-MAPKK Modules and MAPKK-MAPKKK Modules Using Yeast Two-Hybrid (Y2H) Assay

**Figure 5:**
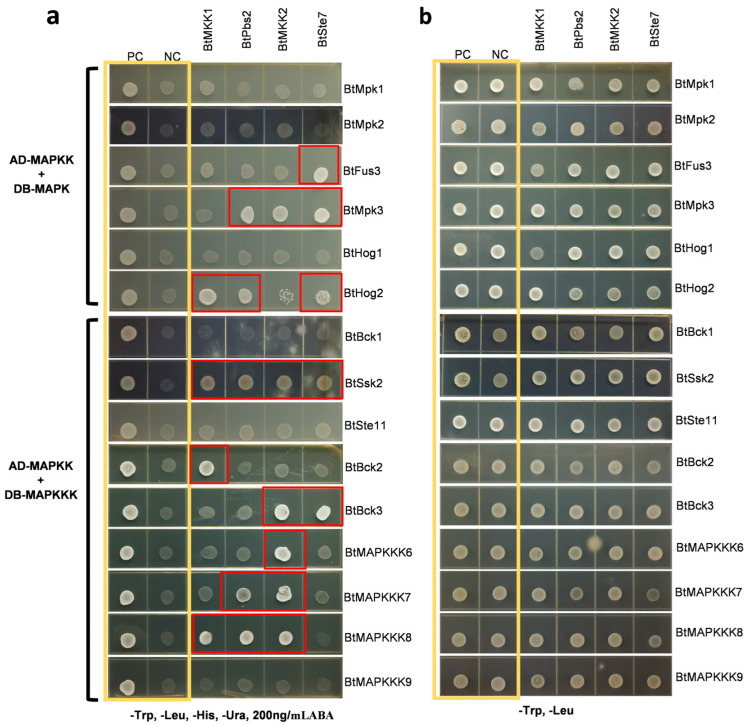
Y2H assays of BtMAPKKs interacting with BtMAPKs or BtMAPKKKs. The yellow boxes are positive and negative controls, while the red boxes indicate positive interactions of the test protein-protein interactions. (**a**) The interactions were screened on SD-His/-Leu/-His/-Ura media with 200 ng/mL AureobasidinA. The AD-T/BD-53 represented a positive control (PC), and AD-T/BD-lam was a negative control (NC). (**b**) All the strains in (**a**) were grown on SD-Trp/-Leu media to be used as the control.

To investigate candidate components in the MAPK protein signaling pathway, six MAPKs and nine MAPKKKs were examined for their interactions with four MAPKKs using a yeast two-hybrid assay. For initial screening, BtMAPKKs were fused with the GAL4 activation domain, while BtMAPKs and BtMAPKKKs with the GAL4 DNA binding domain. Prior validation of BtMAPKKs auto-activation confirmed its absence (Figure S1). Following this, a total of 20 significant protein-protein interactions were identified from 60 combinations of 19 MAPK cascade proteins (Figure 5). In the Y2H screen, each MAPKK was found to have at least one MAPK as an interacting partner. Nevertheless, it is interesting to notice that three MAPKs (BtMpk1, BtMpk2, and BtHog1) were identified as not interacting with any of the MAPKKs. Similar results were observed in the Y2H analysis between MAPKKs and MAPKKKs. Each MAPKK was found to have 2–5 distinct MAPKKKs as interacting partners. BtMKK1 showed interaction with BtHog2 and three distinct upstream MAPKKKs, namely BtSsk2, BtBck2, and BtMAPKKK8. BtPbs2 was found to interact with BtMpk3, BtHog2, BtSsk2, BtMAPKKK7, and BtMAPKKK8. BtMKK2 was shown to form a BtMKK2-BtMpk3 complex and to interact with five different MAPKKKs (BtSsk2, BtBck3, BtMAPKKK6, BtMAPKKK7, and BtMAPKKK8). In the case of BtSte7, three distinct downstream MAPKs, namely BtFus3, BtMpk3, and BtHog2, and two upstream MAPKKs, namely BtSsk2 and BtBck3, were found as interacting proteins. In initial screens, BtSsk2, the homologous protein of ScSsk2, was shown to interact with each BtMAPKKs. To ascertain whether BtSsk2 exhibits autoactivation, BtSsk2-pGBKT7 was examined for its ability to activate reporter genes in the presence of blank vector pGADT7. Furthermore, the interactions between BtPbs2 and BtMPKKKs were later confirmed by swapping the vectors in the Y2H screenings.

### 2.7. Expression Pattern Analysis of BtMAPKs During Mating and Blue Light Conditions

**Figure 6:**
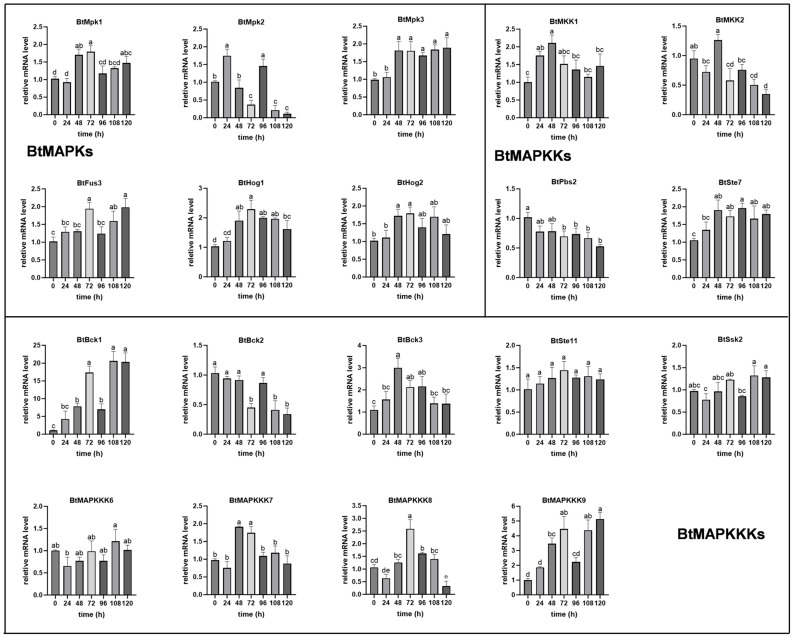
qRT-PCR expression analysis of MAPK cascade family genes in *B. trispora* in response to sexual stimulation conditions. Different superscript letters indicate significant differences at the 0.01 probability level. Data indicate relative expression levels (means ± SE) from three independent biological replica.

Blue light and sex hormones are both important environmental signals for *B. trispora,* as they induce over-accumulation of carotenoids. As highly evolutionarily conserved and key signaling modules in eukaryotes, MAPK cascades contribute to induction-specific signal transduction by phosphorylation and activating their multiple downstream targets. In order to evaluate the contribution of BtMAPK cascade proteins to the response to different signals, we analyzed the transcriptional behavior of *BtMAPKs*, *BtMAPKKs*, and *BtMAPKKKs* in blue light and mating conditions. As shown in Figure 6, the expression levels of *BtMpk1*, *BtMpk3*, *BtHog1*, and *BtHog2* were observed to increase following 48 h of mating, while the expression of *BtMpk2* and *BtFus3* were induced individually at 24 h and 72 h of treatment. Interestingly, the mRNA levels of *BtMpk2* showed a wide range throughout the mating fermentation process. In the case of *MAPKKs*, both *BtMKK1* and *BtSte7* presented higher gene expression levels at 48 h, and then the expression of *BtMKK1* was comparable to that of the control, while *BtSte7* remained at a higher expression level until the end of fermentation. Moreover, the expression levels of *BtPbs2* and *BtMKK2* exhibited a nearly two-fold decrease at 96 h and 108 h, respectively, with no obvious difference observed during the initial stages of mating. Among the nine *BtMAPKKKs*, *BtBck1* was the most abundantly expressed and showed a periodical cyclic expression pattern. The expression level was found to be five-fold higher at 48 h than that of the control, with a substantial maximum increase of 20-fold at 72 h. Thereafter, a decrease to five-fold higher at 96 h was observed, followed by an upregulation to 20-fold again at 120 h. *BtMAPKKK9* and *BtBck1* presented similar expression patterns, except that the highest expression level of *BtMAPKKK9* was 5-fold that of the control during the process of mating. *BtBck3* and *BtMAPKKK7* also showed similar expression patterns. Both of them have shown an increase in transcript levels by 2–3 folds at 48 h compared to the control, before returning to baseline levels. Another member of the BtMAPKKK family, *BtMAPKKK8*, demonstrated a notable elevation in expression levels at 72 h, followed by a pronounced decline at 120 h. In contrast, the transcripts of *BtBck2* exhibited a 2-fold reduction at the 72 h, 108 h, and 120 h time points. In addition, the transcriptional levels of *BtSsk2*, *BtSte11*, and *BtMAPKKK6* showed no difference within 120 h.

**Figure 7:**
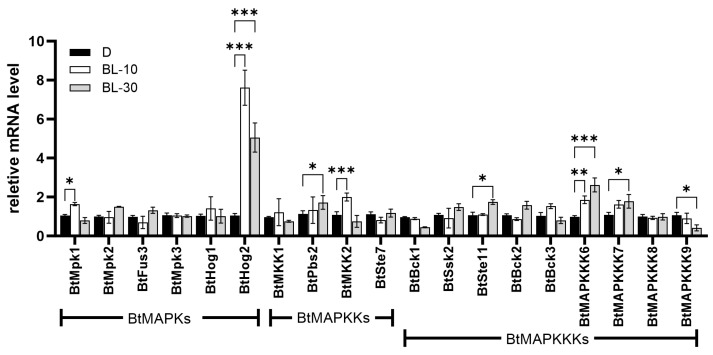
qRT-PCR expression analysis of MAPK cascade family genes in *B. trispora* in response to blue light. The spores were precultured under dark conditions for 48h and then stimulated with blue light for 10 min and 30 min, respectively. Mycelium cultured under dark conditions and irradiated by blue light were harvested and labeled D, BL-10, and BL-30, respectively. * *p*  ≤  0.05, **: *p*  ≤ 0.01, ***: *p*  ≤  0.001 with two-way ANOVA statistical test.

Transcript analysis of all the MAPK pathway genes in *B. trispora* was carried out by quantitative real-time PCR after exposure to blue light for 10 min and 30 min (Figure 7). The majority of these genes exhibited basal expression levels, while seven of them were upregulated and only one gene was downregulated. Among the genes that were upregulated, *BtHog2* has shown the highest transcript level, reaching up to 8-fold that of the control in 10 min, and the transcript levels remained elevated even after 30 min of light exposure, which suggested BtHog2 served as one of the key components in the blue light triggered signal transduction pathway. *BtMAPKKK6* exhibited a continuous increased expression level throughout the irradiation process. The expression levels of *BtMpk1* and *BtMKK2* were increased only after 10 min of blue light irradiation and then returned to normal levels, while *BtPbs2*, *BtSte11*, and *BtMAPKKK7* exhibited suppressed expression. Overall, the transcriptional behaviors of all the MAPK pathway genes were also quite different under the two tested stress conditions.

## 3. Discussion

*B. trispora* can produce large amounts of carotenoids in response to light and sexual signal stimulation, which is thought to be more commercially valuable [27]. However, it remains unclear how it recognizes, transmits, and responds to extracellular signals. In this research, we conducted the first extensive analysis of the MAPK cascade family proteins in *B. trispora*’s signaling network, revealing the coding genes for 6 MAPKs, 4 MAPKKs, and 9 MAPKKKs. These proteins can be categorized into three different clades based on MAPK, MAPKK, and MAPKKK, each of which corresponds to similar cascade proteins from plant origin, according to a phylogenetic analysis of these proteins with MAPK cascade proteins from several representative fungi as well as Arabidopsis. In fungi, the MAPK cascade proteins are more evolutionarily conserved, with MAPKs forming Fus3/Kss1, Hog1, and Slt2/Smk1 subfamilies; MAPKKs creating Mkk1/Mkk2, Ste7, and Pbs2 subfamilies; and MAPKKKs forming Bck1, Ste11, and Ssk2 subfamilies, respectively [23]. The evolutionary conservation of fungal MAPK cascade kinases is also well supported by these investigations based on multiple sequence alignment, conserved motif, and domain analysis.

Genome sequencing and analysis indicated the presence of a rare whole-genome duplication event in the chromosomes of Mucoromycota fungi, despite the fact that gene duplication has played a significant role in the evolution of gene families and the generation of new functions [28]. In the MAPK cascade proteins of *B. trispora*, an estimate of 9 genes for MAPKKKs suggests significant expansion compared to an average of 2–5 genes in Dikarya. Only 4 MAPKKs were identified, which indeed have not duplicated compared to the 2–6 genes identified in Dikarya. Six MAPK proteins are predicted in *B. trispora*, whereas only two MAPKs in *Saccharata proteae* and a maximum of 20 MAPKs in *Laccaria amethystina* from a previous report [22]. Unless the numbers of MAPK gene members in fungi are diverse and vary from species to species, it appears that there is no expansion for the *B. trispora* MAPK gene from the overall count. However, the Hog1 subfamilies and Slt2/Smk1 subfamilies have 2 and 3 copies, respectively, suggesting that they have undergone expansion events. MAPKKK acts upstream of the MAP kinases, implying that MAPKKK’s upstream signaling pathways are more complicated and specialized, allowing them to receive a broader range of signals and transmit them to downstream targets. The upstream signaling pathways of the MAPK cascade, such as G-protein signaling and two-component signal transduction systems, indeed show larger expansions in the *B. trispora* genome and related species [28], which also supports our hypothesis.

The conserved MAPK signaling pathways are involved in sensing and responding to various signals in fungi and are important components of a wide range of fungal life activities, where the sensing and transmission of signals is usually accompanied by changes in the transcriptional levels of the MAPK cascade genes [29]. Bck1, Mkk1/MKK2, and Slt2 are three mitogen-activated protein (MAP) kinases constituting the cell wall integrity (CWI) pathway that mediates the response of *S. cerevisiae* to cell wall alterations [30]. Consequently, we found that BtBck1, a homologous protein of yeast Bck1, exhibited a continuous transcriptional increase from 24 h post-mating, peaking at 20-fold higher than baseline levels. We speculate that when BtBck1 is activated by sex pheromones, positive and negative strains tend to alter cell wall integrity and become more likely to form intersexual heterokaryotic mycelia, exhibiting an increase in the transcriptional level of BtBck1. However, the transcription level of the BtSte11, which is identified as a Ste11 homologue, did not fluctuate throughout the process. The Ste11-Ste7-Fus3 is a classic pheromone response module in yeast, which made the result confusing. Considering that the sexual pheromone of *B. trispora* is trisporic acid, which is a metabolic derivative of β-carotene and has a chemically different structure from the small peptide pheromones of Dikarya fungi, we proposed that the sexual pheromone response pathway of *B. trispora* is distinct from that of the Dikarya fungi. Additionally, it is noteworthy that BtMAPKKK9, which belongs to the same evolutionary branch as AtMAPKKK6 and AtMAPKKK7 in Arabidopsis (Figure 1), showed a 3.5-fold increase in transcription level in 48 h. It led us to speculate the possibility that the response of *B. trispora* to sex pheromones may be similar to that of Arabidopsis to plant hormones. However, the AtMAPKKK6 and AtMAPKKK7 signaling pathways have not been studied yet in Arabidopsis. Coincidentally, abscisic acid, one of the well-known apocarotenoid plant hormones, has been used as a structural analog of trisporic acid for lycopene or β-carotenoid production by mated fermentation of *B. trispora*, and it exerted similar effects to trisporic acid [31]. This fact provided evidence for the similarity of MAPK signal pathways in response to apocarotenoid. In blue light conditions, BtMAPK6, which belongs to the Hog1 subfamily, exhibits a significant increase in transcription level, indicating that the Hog1 pathway is involved in the blue light signal transduction in *B. trispora*. According to the investigations in other fungi, Hog1 has been confirmed to participate in blue and red light signal transduction and light regulation of secondary metabolite biosynthesis by interacting with various proteins [32,33,34]. By comprehensively analyzing this finding and previous research on *B. trispora* photoreceptors WC-1/WC-2, it can be inferred that the MAPK cascade pathway is functionally more conserved in light signal response across the fungal kingdom. In contrast, functions of the MAPK cascade pathways are significantly different in the response to pheromones between early-diverged fungi and dikarya fungi, possibly due to the differences in strategies in sexual reproduction and sex pheromone structure.

Through in silico approaches, we predicted 16 potential BtMAPKKK-BtMAPKK and 12 BtMAPKK-BtMAPK interactions. However, yeast two-hybrid (Y2H) assays detected 13 BtMAPKKK-BtMAPKK interactions and 7 BtMAPKK-BtMAPK interactions (Table 2). Notably, while computational models predicted 28 interactions, experimental validation confirmed only partial overlap (11/28). These discrepancies between computational predictions and experimental results likely stem from structural inaccuracies in AlphaFold-derived 3D models used for docking, which fail to account for phosphorylation-induced conformational changes in vivo, thereby emphasizing the necessity of in vivo context for pathway reconstruction.

**Figure 8:**
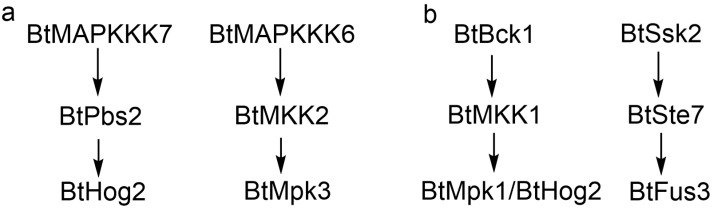
MAPK cascade signaling pathways in *B. trispora* based on Y2H assays, in-silico predictions, and RT-PCR expression analysis. (**a**) Response to light. (**b**) Response to sexual stimulation.

The integrated signaling network proposed in Figure 8 synthesizes experimental and computational evidence to delineate distinct MAPK cascade modules governing light and sexual signaling in *B. trispora*. For light response (Figure 8a), BtHog2 exhibited rapid transcriptional activation (8-fold upregulation at 10-min blue light) and sustained expression, corroborating its role as a central mediator of photoadaptation. The predicted interaction between BtHog2 and BtPbs2 (MAPKK) was supported by both Y2H assays and molecular docking. Concurrently, BtMAPKKK7 showed elevated transcription under light and interacted with BtPbs2, suggesting a putative BtMAPKKK7-BtPbs2-BtHog2 cascade. Notably, the absence of BtHog1 interaction with MAPKKs suggests functional divergence within the Hog1 subfamily, potentially reflecting niche-specific adaptations in light sensing. In addition, BtMAPKKK6 demonstrated progressive transcriptional upregulation during light irradiation, forming a potential BtMAPKKK6-BtMKK2-BtMpk3 pathway via Y2H-confirmed interactions. In sexual signaling, the expression analysis suggested the pheromone response network (Figure 8b) highlights BtBck1 (MAPKKK) as a key regulator. Although Y2H failed to detect BtBck1-MAPKK interactions, molecular docking suggested its binding to BtMKK1, which relays signals to BtMpk1 and BtHog2 (Table 2), forming a BtBck1-BtMKK1-BtHog2/Mpk1 cascade. Intriguingly, Y2H revealed a non-canonical BtSsk2-BtSte7-BtFus3 signaling pathway in *B. trispora*, which diverges from the conserved Fus3/Kss1 mating module observed in *S. cerevisiae*. Combined with the absence of detectable interactions between BtSte11 and MAPKKs, these findings demonstrate evolutionary rewiring of sexual signaling pathways in *B. trispora*, likely driven by structural divergence of trisporic acid from Dikarya pheromones. Collectively, Figure 8 provides a scaffold for probing lineage-specific MAPK dynamics, though further validation via phosphoproteomics and genetic manipulation is critical to resolve mechanistic ambiguities.

These results have given us a preliminary understanding of the MAPK cascade network in *B. trispora* and provided us some valuable clues. To gain more insight into this signaling network, it is necessary to conduct additional experiments such as gene knockout, gene editing, and RNA interference. However, one limitation of these methods is that the genetic manipulation techniques in this strain are still not fully developed. Therefore, one important future direction is to establish a comprehensive and systematic set of genetic manipulation tools to facilitate thorough gene function research.

## 4. Materials and Methods

### 4.1. Strains, Medium, and Culture Conditions

*B. trispora* NRRL2896(−) and NRRL2895(+) were selected for this study. Spores were harvested from mycelia grown for 5 days on YpSs agar medium [3] and kept in glycerol (40% *w/v* in water) at 4 °C. For mated culture, these strains were first cultivated separately. A total of 1 × 10^6^ spores were inoculated into 500-mL flasks containing 100 mL of fermentation medium and cultured at 25 °C for 3d. Subsequently, the mycelium was collected and transferred to a new 500-mL flask containing 100 mL of fermentation medium. For blue light stimulation, 10^4^ spores of NRRL2896(−) were inoculated in PDA plates and cultivated in the dark for 48 h. The plates were then irradiated by blue light at 1.34 W/m^2^ for 10 min and 30 min. The mycelia were collected and then frozen in liquid nitrogen immediately. *Escherichia coli* DH5α was used for cloning plasmids, and yeast strain Y2H Gold was used for the yeast two-hybrid assay.

### 4.2. Gene Discovery and Bioinformatic Analysis of BtMAPK Cascade Proteins

To identify MAPK cascade proteins within the proteome of *B. trispora*, the 13 MAPK cascade protein sequences (5 genes encoding MAPKs, 4 MAPKKs, and 4 MAPKKKs) of *Saccharomyces cerevisiae* and 110 MAPK cascade protein sequences (20 genes encoding MAPKs, 10 MAPKKs, and 80 MAPKKKs) of *A. thaliana* were used as the search query. The BlastP 2.2 program was used to identify the MAPK cascade protein sequences from the *B. trispora* genome database (https://mycocosm.jgi.doe.gov/Blatri1/Blatri1.home.html, accessed on 13 January 2024). The applied E value threshold was 10^−8^. The online software Pfam 37.3 (http://pfam.sanger.ac.uk/search, accessed on 7 November 2024), SMART 8.0 (http://smart.embl-heidelberg.de/, accessed on 7 November 2024), and NCBI Conserved Domain Search web tools (http://www.ncbi.nlm.nih.gov/Structure/cdd/wrpsb.cgi, accessed on 7 November 2024) were used to check for the presence of serine/threonine protein kinase domain.

Physicochemical Parameters such as length of sequences, isoelectric point (pI), and molecular weight (MW) of each gene product were evaluated using the protParam tool from the ExPASy online service (http://web.expasy.org/protparam/, accessed on 28 October 2024) [35]. The subcellular localization prediction was performed using ProtComp 9.0 from Softberry (http://www.softberry.com, accessed on 28 October 2024). A phylogenetic analysis based on the full-length protein sequences was performed using MEGA X software, and the phylogenetic tree was constructed using the neighbor-joining (NJ) method and testing with 1000 bootstrap replicates [36]. Modification of the evolutionary tree was done using the online tool Evolview-v2. The MAPK cascades protein sequences were subjected to the online tool MEME for conserved motif analysis (https://meme-suite.org/meme/tools/meme, accessed on 28 October 2024) [37]. Conserved domains were identified using the InterProScan database. The gene structures based on the GFF annotation file of the *B. trispora* genome were analyzed by Gene Structure Display Server (GSDS) 2.0 (http://gsds.cbi.pku.edu.cn, accessed on 28 October 2024) [38]. TBtools v2.113 was used to merge the gene structure, protein conserved motifs, protein structural domains, and phylogenetic tree of the MAPK cascades family. Multiple sequence alignment was done using the ClustalW 2.1 and then was imported to GeneDoc 2.7 for display.

### 4.3. RNA Extraction and Quantitative RT-PCR

The mycelia of *B. trispora* treated with sexual interaction and blue light were used to isolate total RNA. RNA extraction was performed using the EasyPure^®^ Plant RNA Kit (Transgen, Shanghai, China) according to the manufacturer’s instructions. First-strand cDNA was subsequently synthesized from total RNA using EasyScript^®^ First-Strand cDNA Synthesis SuperMix (Transgen, Shanghai, China). The quality of total RNA was verified through agarose gel electrophoresis.

For the analysis of quantitative real-time PCR (qRT-PCR), specific primer pairs for 19 MAPK cascade family genes were designed with Primer Premier v5.0 software (Table S3). The amplicon product of each of them was around 200 bp. qRT-PCRs were performed on a LightCycler^®^ 96 instrument (Roche, Basel, Switzerland) with TB Green^®^ Premix Ex Taq^™^ II FAST qPCR (Takara, Tokyo, Japan). PCR reactions were performed in triplicate as per the provided instructions. The amplification program used in this study consisted of 95 °C for 3 min; 40 cycles of 95 °C for 10 s and 60 °C for 20 s; and a melting curve of 60 °C to 95 °C at increments of 0.5 °C for 10 s. Data analysis for each gene was performed using the comparative CT method (ΔΔCT). The endogenous GAPDH gene was used as the control for normalization. Three biological replicates were performed for each experiment.

### 4.4. Yeast Two-Hybrid (Y2H) Assay

The coding sequences of all the 19 MAPK cascade family proteins were amplified via RT-PCR using the gene-specific primers listed in Table S3. The BtMAPKK genes were cloned into the pGADT7 vector, while BtMAPK and BtMAPKKK genes were cloned into pGBKT7. The plasmid pairs were co-transformed into the yeast strain Y2H Gold, which was then grown at 30 °C on synthetic defined (SD) medium lacking leucine and tryptophan. The bait auto-activation tests were performed on SD medium without Trp and Leu but supplemented with different concentrations of aureobasidin A (AbA; 0–300 ng/mL). SD-dropout medium (SD/-Trp/-Leu/-His/-Ade) containing 200 ng/mL AbA was used for interaction screening.

### 4.5. Prediction of Protein Structure and Molecular Docking

The tertiary structures of all the MAPK cascade proteins were predicted by AlphaFold2 (https://colab.research.google.com/github/sokrypton/ColabFold/blob/main/AlphaFold2.ipynb, accessed on 30 January 2024) and the overall prediction process was as described in the AlphaFold paper [39]. Amongst five AlphaFold provided models for each protein, those ranking first were used for protein-protein docking inputs. Docking analysis was performed using the ZDOCK 3.0.2 and RDOCK 2022.0+1 programs [40]. A set of docked protein poses were generated by ZDOCK and ranked with the ZDOCK score. The ZRank score is calculated using the energy of the initial-stage docked poses for reranking them. RDOCK was used to generate low-energy poses during the refinement stage of protein docking. The results were ranked based on E_RDOCK. Docking poses that have low E_RDOCK energies correspond to near-native structures. The PDB viewer was used for the visualization of structures showing the interactions between molecules.

## Figures and Tables

**Table 1:** Characteristics of the predicted BtMAPK cascade proteins in *B. trispora*.

No.	Family	MAPK Names	Alter Names	Protein_ID	Amino Acid Size	PI	Mw (kDa)
1	MAPK	BtMAPK1	BtMpk1	Bt_461469	491	5.62	56,014.63
2		BtMAPK2	BtMpk2	Bt_414654	489	6.18	56,149.88
3		BtMAPK3	BtFus3	Bt_459063	352	6.02	40,932.31
4		BtMAPK4	BtMPK3	Bt_488485	432	5.72	49,237.41
5		BtMAPK5	BtHog1	Bt_177194	384	5.21	43,579.11
6		BtMAPK6	BtHog2	Bt_484059	375	5.27	42,769.40
7	MAPKK	BtMAPKK1	BtMKK1	Bt_474273	306	5.45	34,445.05
8		BtMAPKK2	BtPbs2	Bt_486874	417	6.15	46,153.97
9		BtMAPKK3	BtMKK2	Bt_472095	349	5.16	38,864.24
10		BtMAPKK4	BtSte7	Bt_482330	314	6.15	35,225.52
11	MAPKKK	BtMAPKKK1	BtBck1	Bt_489889	803	7.73	91,348.49
12		BtMAPKKK2	BtSsk2	Bt_421286	1327	5.39	151,528.36
13		BtMAPKKK3	BtSte11	Bt_375242	829	6.15	93,010.83
14		BtMAPKKK4	BtBck2	Bt_383446	1264	6.57	140,937.58
15		BtMAPKKK5	BtBck3	Bt_515500	873	5.86	99,000.54
16		BtMAPKKK6	BtMAPKKK6	Bt_375536	259	5.90	28,496.7
17		BtMAPKKK7	BtMAPKKK7	Bt_470289	723	6.21	81,594.52
18		BtMAPKKK8	BtMAPKKK8	Bt_433238	516	5.71	57,720.06
19		BtMAPKKK9	BtMAPKKK9	Bt_484622	743	9.01	83,919.86

**Table 2:** Direct comparison of yeast two-hybrid assay and computational dockings with respect to *B. trispora* MAPKKK-MAPKK and MAPKK-MAPK interaction.

MAPKK	Interacting MAPKs/MAPKKKs Predicted by Docking Study	Interacting MAPKs/MAPKKKs Identified by Y2H Screen
BtMKK1	BtMpk1, **BtHog2**	**BtHog2**
	BtBck1, BtSte11, **BtBck2,** BtMAPKKK7, BtMAPKKK9	BtSsk2, **BtBck2**, BtMAPKKK8
BtPbs2	BtFus3, BtHog1, **BtHog2**	BtMpk3, **BtHog2**
	**BtSsk2**, BtSte11, **BtMAPKKK7**, BtMAPKKK9	**BtSsk2**, **BtMAPKKK7**, BtMAPKKK8
BtMKK2	BtMpk1, BtFus3, BtHog1	BtMpk3
	**BtSsk2**, **BtMAPKKK6**, **BtMAPKKK7**	**BtSsk2**, BtBck3, **BtMAPKKK6**, **BtMAPKKK7**, BtMAPKKK8
BtSte7	BtMpk1, **BtMpk3**, BtHog1, **BtHog2**	BtFus3, **BtMpk3**, **BtHog2**
	**BtSsk2**, BtSte11, BtMAPKKK7, BtMAPKKK9	**BtSsk2**, BtBck3

The MAPKs/MAPKKKs interacting with MAPKK, identified concurrently through Y2H and in silico prediction, are shown in bold.

## Data Availability

The data presented in this study are available on request from the corresponding author.

## Supplementary Materials

The following supporting information can be downloaded at: https://www.mdpi.com/article/10.3390/ijms26104789/s1.
