# Supplementary figures and images for: Genome-Wide Identification, Phylogeny and Expressional Profiles of Mitogen Activated Protein Kinase Gene Family in Blakeslea trispora
Source: Int J Mol Sci. 2025 May 16;26(10):4789. doi: 10.3390/ijms26104789 (PMC12111849; doi:10.3390/ijms26104789)

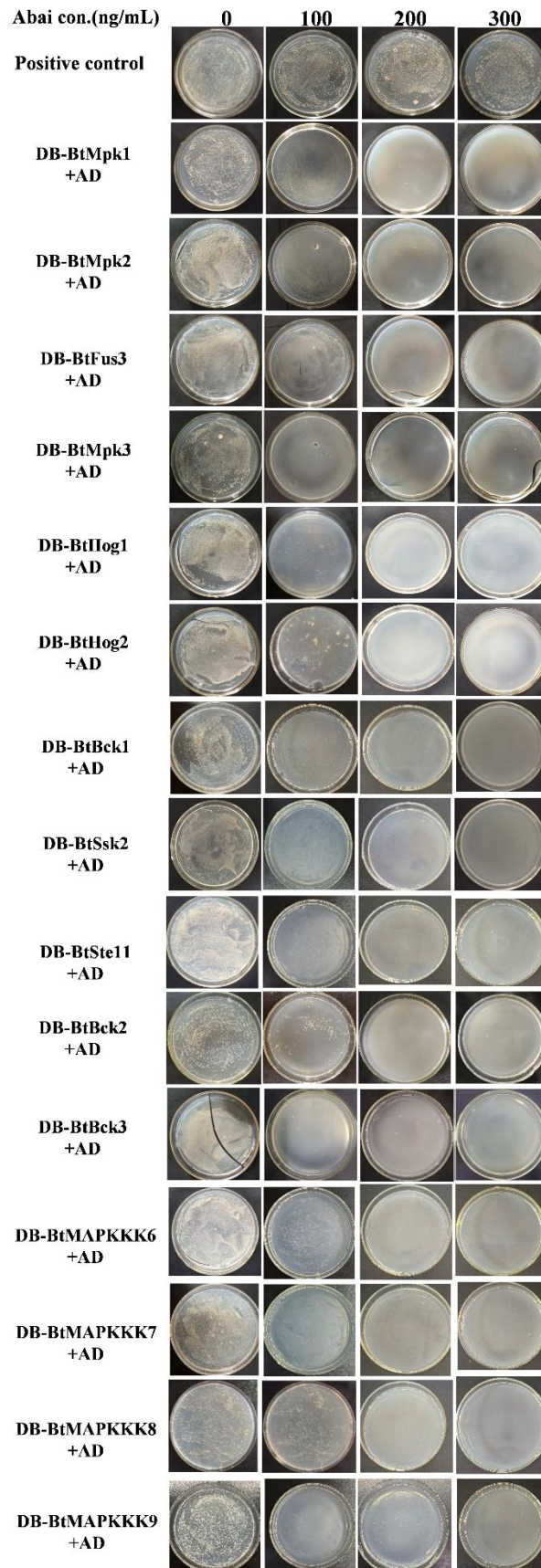

**Figure S1.** Bait auto-activation tests of Y1H screens.

Supplement: Supplementary file 1 [file ijms-26-04789-s001.zip › Supplementary figure S1.pdf]
